# First Report on the Chemical Composition, Antioxidant Capacity, and Preliminary Toxicity to *Artemia salina* L. of *Croton campinarensis* Secco, A. Rosário & PE Berry (Euphorbiaceae) Essential Oil, and In Silico Study

**DOI:** 10.3390/antiox11122410

**Published:** 2022-12-06

**Authors:** Leonardo Souza da Costa, Ângelo Antônio Barbosa de Moraes, Jorddy Neves Cruz, Suraj N. Mali, Lorena Queiroz Almeida, Lidiane Diniz do Nascimento, Oberdan Oliveira Ferreira, Everton Luiz Pompeu Varela, Sandro Percário, Mozaniel Santana de Oliveira, Eloisa Helena de Aguiar Andrade

**Affiliations:** 1 School of Chemical Engineering, Institute of Technology, Universidade Federal do Pará, Belem 66075-110, Brazil; 2 Adolpho Ducke Laboratory, Coordination of Botany, Emílio Goeldi Museum of Pará, Belem 66077-830, Brazil; 3 Laboratory of Functional and Structural Biology, Institute of Biological Sciences, Universidade Federal do Pará, Belem 66075-110, Brazil; 4 Department of Pharmacy, Government College of Pharmacy, Affiliated to Shivaji University, Kolhapur, Karad 415124, Maharashtra, India; 5 Oxidative Stress Research Laboratory, Biological Sciences Institute, Universidade Federal do Pará, Belem 66075-110, Brazil

**Keywords:** Amazon, new species, natural products, bioactive compounds, molecular modeling

## Abstract

*Croton campinarensis* Secco, A. Rosário & PE Berry is an aromatic species recently discovered in the Amazon region. This study first reports the chemical profile, antioxidant capacity, and preliminary toxicity to *A. salina* Leach of the essential oil (EO) of this species. The phytochemical profile of the essential oil was analyzed by gas chromatography (GC/MS) and (GC-FID). The antioxidant capacity of the EO was measured by its inhibition of ABTS^•+^ and DPPH^•^ radicals. Molecular modeling was used to evaluate the mode of interaction of the major compounds with acetylcholinesterase (AChE). The results indicate that the EO yield was 0.24%, and germacrene D (26.95%), bicyclogermacrene (17.08%), (*E*)-caryophyllene (17.06%), and δ-elemene (7.59%) were the major compounds of the EO sample. The EO showed a TEAC of 0.55 ± 0.04 mM·L^−1^ for the reduction of the ABTS^•+^ radical and 1.88 ± 0.08 mM·L^−1^ for the reduction of the DPPH^•^ radical. Regarding preliminary toxicity, the EO was classified as toxic in the bioassay with *A. salina* (LC_50_ = 20.84 ± 4.84 µg·mL^−1^). Through molecular docking, it was found that the majority of the EO components were able to interact with the binding pocket of AChE, a molecular target related to toxicity evaluated in *A. salina* models; the main interactions were van der Waals and π-alkyl interactions.

## 1. Introduction

Natural products, in particular volatile oils isolated from aromatic plants, have been the subject of several studies over the years. This may be related to their complex chemical compositions, which may include a series of classes of chemical compounds such as monoterpenes, sesquiterpenes, hydrocarbons, oxygenated mono- and sesquiterpenes, and phenylpropanoids [1,2,3,4]. In addition, this diverse chemical composition may be related to potential activities, such as antioxidant [5,6,7]. Amazonian flora, for example, include several aromatic plant species that produce EOs [8,9] that can serve as inputs for various sectors of the chemical, pharmaceutical, cosmetic, and food industries; these potential applications show promise in generating development in the region [4,10,11,12]. Among Amazonian species that produce EOs, those belonging to the family Euphorbiaceae stand out, especially species of the genus *Croton* [13].

The *Croton* genus includes a variety of over 1200 species with a widespread global distribution in both the tropics and subtropics, especially in arid and semiarid zones [14]. In Brazil, 300 species are found, of which 230 are considered endemic. In the Amazon region, 61 species have been recorded [15]. *Croton* species have shrub characteristics, can resprout in rainy seasons, and grow widely, especially in deforested areas [16].

Several species of *Croton* have chemical compounds used for medicinal purposes, mainly as anti-inflammatory, antihypertensive, antifungal, antimicrobial, antidiabetic, antioxidant, antinociceptive, and antitumor agents [16]. In traditional medicine, the leaves of species of this genus are used to treat gastrointestinal disorders, rheumatism, migraine, diabetes, cholesterol level, inflammatory diseases, and bronchitis [17,18,19]. The EOs of species of the genus *Croton* are characterized by different chemical classes of compounds, with a predominance of terpenoids and phenylpropanoids [20]. Compounds such as limonene, (*E*)-caryophyllene, spathulenol, bicyclogermacrene, germacrene D, (*E*)-anathole, and estragol are common components of EOs of *Croton* species [21,22,23,24,25,26].

The EOs of *Croton* species have also been widely studied for the discovery of new natural antioxidants [27,28,29,30,31]. Antioxidant substances can inhibit free radicals [32,33], which may be responsible for the damage caused by oxidative stress related to various diseases, such as Alzheimer’s disease, Parkinson’s disease, cancer, and diabetes [34]. The use of natural antioxidant compounds rather than synthetic antioxidants is currently being widely explored, as the former pose lower human health risks, especially those related to high toxicity and the triggering of new diseases caused by synthetic products [35,36,37].

When evaluating the toxicity of EOs, preliminary tests are performed to ensure their safety for humans [38,39]. Preliminary toxicity tests on *A. salina* allow an initial response to the potential toxicity of an extract or isolated substance; this is due to the sensitivity similar to that of human cells that the microcrustacean presents. [40]. The EOs of some species of the genus *Croton* have moderate or high toxicity against *A. salina*, which is directly related to the high sesquiterpene content in their chemical composition [41,42,43,44].

This bioassay is also important due to the biochemical activity of the enzyme acetylcholinesterase (AChE), which mediates the larval mortality of *A. salina* when individuals come into contact with EO within 24 h; the number of deaths of microcrustaceans is counted, which indicates a potential biological activity of the essential oil [45]. This enzyme is also directly related to the behavior and physiology of *A. salina,* and its inhibition may cause deleterious effects in individuals [46].

*Croton campinarensis* Secco, A. Rosário & PE Berry is a recently discovered species recorded for the first time in 2012 [47]. The species is listed only in the state of Pará, located in the Brazilian Amazon [15]. Because it is a recently known species, there are no records in the literature related to the chemical composition and antioxidant and biological properties of its EO. As a result, the present study first reports the yield, chemical composition, antioxidant profile, and preliminary toxicity of the EO of dry leaves of *C. campinarensis*, aiming to contribute to the phytochemical knowledge of aromatic plants of the genus *Croton* from the Amazon region.

## 2. Materials and Methods

### 2.1. Collection and Processing of Botanical Material

Leaves of *C. campinarensis* were collected in the locality of Campina do Guajará, municipality of Bujaru (Latitude: 1°31′15″ S, Longitude: 48°2′37″ W), microregion of Castanhal, Pará State, Brazil, in July 2017. The sample was identified and deposited in the herbarium of the Museu Paraense Emílio Goeldi, Belém, Pará, with registration number MG167619.

### 2.2. Distillation of Essential Oil

The processed botanical material was subjected to hydrodistillation to obtain EO, using a modified Clevenger apparatus for 3 h. After distillation, the EO was centrifuged and dehydrated with anhydrous sodium sulfate (Na_2_SO_4_). Then, it was stored and preserved in a freezer. The EO yield was calculated on a moisture-free basis [45].

### 2.3. Analysis of the Chemical Composition of the Essential Oil

The analysis of the phytochemical profile of the essential oil of *C. campinarensis* was carried out following the same protocols of our research group, as well as the brand and model of the equipment [33,48,49,50]. Masses (GC-MS) and quantification were performed by gas chromatography with a flame ionization detector (GC-FID). The identifications in the GC/MS were performed based on the calculated retention index [51] and compared with the literature [52].

### 2.4. Determination of the Trolox Equivalent Antioxidant Capacity (TEAC) of the Essential Oil

#### 2.4.1. DPPH Method

This method was performed to analyze the potential of EO of *C. campinarensis* to inhibit the 1,1-diphenyl-2-picrylhydrazyl (DPPH^•^) radical, a violet chromophore, resulting in the formation of the hydrogenated DPPH product, which is yellow or colorless [53]. Description of the method can be found in [54].

#### 2.4.2. ABTS Method

This method was performed to analyze the potential of *C. campinarensis* EO to inhibit the 2,2-azino-bis (3-ethylbenzothiazoline-6-sulfonic acid) diammonium salt (ABTS; Sigma-Aldrich; A1888, São Paulo, Brazil) radical, according to modification by Re et al. [55] of the experimental method proposed by Miller et al. [56]. Description of the method can be found in [54].

### 2.5. Determination of Preliminary Toxicity against Artemia salina Leach

For toxicity tests on *A. salina*, the essential oil was prepared at concentrations ranging from 1–100 μg·mL^−1^, according to methods described by [45]. A total of ten *A. salina* larvae were added to each test flask with the aid of automatic micropipettes. In the control group and the positive group with lapachol, the same solvent was used for the samples and larvae, under the same conditions as the bioassay. The counting of *A. salina* was carried out after a period of 24 h at each concentration used and the IC50 was calculated, with the experiments being carried out in triplicate (*n* = 3).

### 2.6. Statistical Analysis

In the experimental tests, with the exception of the analysis of the chemical composition, the statistical Student’s *t*-test was applied, and a significance level of 5% (*p* ≤ 0.05) was considered.

### 2.7. Molecular Docking

For the molecular docking studies, data on the major compounds germacrene D (26.95%), bicyclogermacrene (17.98%), (*E*)-caryophyllene (17.60%), γ-terpinene (8.99%), and δ-elemene (7.59%) were obtained from the PubChem database (https://pubchem.ncbi.nlm.nih.gov/, accessed on 1 October 2022). Then, their structures were optimized with B3LYP/6-31G* using Gaussian 09 software (Gaussian, Inc., Wallingford, England) [57,58,59,60].

Molecular interactions were performed with the majority compounds and AChE in Molegro Virtual Docker (MVD) 5.5 software (Molexus IVS, Odder, Denmark) [61,62,63,64]; the structure of the protein used in the molecular modeling study can be obtained from the Protein Data Bank (https://www.rcsb.org/, accessed on 1 September 2022), using the ID code 4M0E [65]. The MolDock Score (GRID) was carried out as described by [45].

## 3. Results and Discussion

### 3.1. Yield and Chemical Composition of the Essential Oil

The EO yield for the studied sample was 0.24%, we can observe in Table 1. There are no records in the literature regarding the yield and volatile composition of the EO of *C. campinarensis*. However, the yield found in the present study is lower than that reported by Turiel et al. [66] for four *Croton* species from the Amazon (0.50% ± 1.10%). Another notable finding is the high concentration of sesquiterpene hydrocarbons found in both the present sample and the species analyzed by Turiel et al. [66], which had levels between 55.30% and 83.00%.

Regarding the chemical composition, we can observe in Table 1, 32 volatile constituents were identified, accounting for 99.86% of the total, and sesquiterpene hydrocarbons dominated (87.95%), mainly germacrene D (26.95%), bicyclogermacrene (17.08%), (*E*)-caryophyllene (17.06%), and δ-elemene (7.59%). In addition, the monoterpene hydrocarbon γ-terpinene (8.99%) had a high content. Turiel et al. [66] reported that (*E*)-caryophyllene was the major component of the EOs of *C. campestris* (23.90%) and *C. eriocladus* (24.10%), bicyclogermacrene was the main volatile compound of the EO of *C. chaetocalyx* (13.90%), and spatulenol was the component with the highest content in *C. glandulosus* EO (19.70%). The authors also found germacrene D at high levels in the EOs of *C. campestris* (13.70%)*, C. eriocladus* (9.70%), and *C. chaetocalyx* (17.90%); (*E*)-caryophyllene in the EOs of *C. eriocladus* (7.10%) and *C. glandulosus* (8.90%); bicyclogermacrene in the EO of *C. glandulosus* (9.60%); and δ-elemene in the EOs of *C. chaetocalyx* (13.50%) and *C. glandulosus* (8.00%). The authors also noted that γ-terpinene was identified only in *C. campestris* EO, with a low content (0.70%). These results indicate that the present sample of *C. campinarensis* EO has a chemical composition similar to those of other species of this genus present in the Amazon region.

Germacrene D, the major constituent of the sample, shows larvicidal activity against the mosquito *Aedes aegypti* [68]. EOs containing this compound as a major component also have anti-inflammatory and anti-AChE properties [69]. (*E*)-Caryophyllene also has potential anti-inflammatory activity and can be used to treat central nervous system diseases, cancer, and dental caries infections caused by etiological agents [70].

Franco et al. [33] reported that germacrene D and (*E*)-caryophyllene have antioxidant activity, and bicyclogermacrene has been associated with larvicidal and antiviral activity. Figueiredo et al. [71] reported that EOs with high levels of γ-terpinene have moderate activity against food pathogens. According to Dang et al. [72], δ-elemene has anticancer activity against HeLa cells.

Da Silva Júnior et al. [73] reported that germacrene D, bicyclogermacrene and δ-elemene are directly related to plant defense mechanisms and have higher concentrations in the rainy season in the Amazon region. According to the authors, this concentration trend may be directly related to a plant strategy for attracting pollinating agents, especially bees and flies, which are common in the rainy season.

### 3.2. Antioxidant Capacity and Preliminary Toxicity of the Essential Oil

The Table 2, below shows the TEAC of the EO of *C. campinarensis*, measured through the inhibition of ABTS^•+^ and DPPH^•^ radicals and its preliminary toxicity against *A. salina*.

The TEAC of the EO to inhibit the ABTS^•+^ radical was 0.55 ± 0.04 mM·L^−1^ (*p* = 0.557). In the DPPH assay, the TEAC was 1.88 ± 0.08 mM·L^−1^ (*p* = 0.001). These results indicate that the ABTS^•+^ radical capture potential of the EO of *C. campinarensis* was lower than that presented by the Trolox standard (1 mM·L^−1^). On the other hand, for the inhibition of DPPH^•^ radicals, the TEAC of the EO was almost double that of the Trolox standard (1 mM·L^−1^). Regarding the preliminary toxicity against *A. salina*, the EO had an LC_50_ of 20.84 ± 4.84 µg·mL^−1^. According to Ramos et al. [74], EOs with LC_50_ values lower than 80 µg·mL^−1^ are classified as toxic.

There are no reports in the literature regarding the antioxidant capacity and preliminary toxicity of *C. campinarensis* EO. However, other *Croton* species do have literature data on these properties. Morais et al. [75] reported that the evaluation of antioxidant activity by the DPPH method showed that *C. campinarensis* EOs have moderate antioxidant activity. According to the authors, the Croton EOs did not contain phenolic compounds, which is the main cause of their lower antioxidant activity than the phenolic compound thymol and the commercial antioxidant BHT. In addition, the authors identified high levels of oxygenated sesquiterpenes in the samples, especially caryophyllene oxide and spathulenol. Pino et al. [76] found that the EO of *Croton wagneri* from Ecuador had a moderate elimination effect in the DPPH and ferric reducing antioxidant power (FRAP) assays. According to the authors, *cis*-chrysanthenol (27.5%) and myrcene (19.2%) were the major components of the sample.

Do Vale et al. [77] analyzed the antioxidant capacity of the EO of *Croton piauhiensis*, characterized by compounds such as (*E*)-caryophyllene (21.58%), γ-terpinene (10.08%), and germacrene D (9.56%). The authors indicated that this EO showed high antioxidant capacity in the DPPH test, with higher results than the positive control (quercetin).

Regarding preliminary toxicity, Ribeiro et al. [78] evaluated the preliminary toxicity of the EO of *Croton rudolphianus* leaves by bioassays with *A. salina*. According to the authors, this EO exhibited high toxicity to microcrustaceans (LC_50_ = 68.33 µg·mL^−1^). In addition, the authors identified chemical constituents consistent with those found in the present sample, such as (*E*)-caryophyllene (17.33%), bicyclogermacrene (7.1%), and germacrene D (5.38%).

Andrade et al. [79] reported that the EO of *Croton zehntneri* leaves showed high toxicity against *A. salina*, with an LC_50_ of 4.54 µg·mL^−1^. However, the authors found that phenylpropanoid estragol was the major compound in the sample (84.70%). Lawal et al. [80] reported that the EO of *Croton gratissimus* showed toxicity against *A. salina*, with an LC_50_ of 8.52 mg·mL^−1^, corresponding to a classification of toxic. Regarding the chemical composition of the EO, the authors indicated that α-phellandrene (12.30%), β-phellandrene (10.70%), α-pinene (6.05%), and germacrene D (5.90%) were the major components.

Regarding the possible antioxidant capacity of the major components of the EO of *C. campinarensis*, EOs containing germacrene D, bicyclogermacrene, (*E*)-caryophyllene, γ-terpinene, and δ-elemene at high levels have shown relevant activity, scavenging ABTS^•+^ and DPPH^•^ radicals [81,82,83,84,85]. In addition, γ-terpinene can increase the protection of lipids and oxidizable substrates, in addition to prolonging the protective activity of the synthetic antioxidant α-tocopherol, making it a promising natural antioxidant for use in foods [86,87].

Regarding the toxicity of major compounds, Judzentienė et al. [88] reported that the EO of *Artemisia* vulgaris, containing high levels of germacrene D (10.60–30.50%), showed high toxicity against *A. salina*. The authors directly attributed the results to the presence of germacrene D in the sample. Machado et al. [89] reported that (*E*)-caryophyllene showed toxicity against *A. salina* only at high concentrations (3 mM). Schmitt et al. [90] showed that this sesquiterpene had no significant toxicity against rats.

Govindarajan et al. [91] stated that bicyclogermacrene showed high toxicity in mosquitoes of the species *Anopheles subpictus*, *Anopheles albopictus*, and *Culex tritaeniorhynchus*. De Oliveira et al. [92] found that the EO of *Lantana montevidensis* leaves, characterized by germacrene D (31.27%) and (*E*)-caryophyllene (28.15%), showed no fumigant toxicity in *Drosophila melanogaster* flies. EOs containing γ-terpinene and δ-elemene at high levels showed low toxicity against *A. salina* [93,94].

The biological properties of an EO may be associated with the major constituent of the sample and/or the synergistic and antagonistic effects exerted by all components present in the mixture [95]. Sesquiterpenes have higher toxicity than monoterpenes and phenylpropanoids [96]. Regarding free radical capture ability, sesquiterpenes generally have a lower antioxidant capacity than monoterpenes and phenylpropanoids [97]. These attributes may explain the antioxidant profile and preliminary toxicity exhibited by the EO of *C. campinarensis.*

### 3.3. Analysis of the Interactions of Major Compounds with AChE

In silico methods have been successfully used to evaluate the interaction between molecules of natural origin and molecular targets of pharmacological interest [98,99,100,101]. In this study, molecular docking was used to evaluate how the major compounds of *C. campinarensis* interact with the binding pocket of AChE, a molecular target related to toxicity that is investigated in *A. salina* models [45]. The energy values obtained for the interactions of the compounds with the target enzyme are summarized in Table 3.

**Figure 1:**
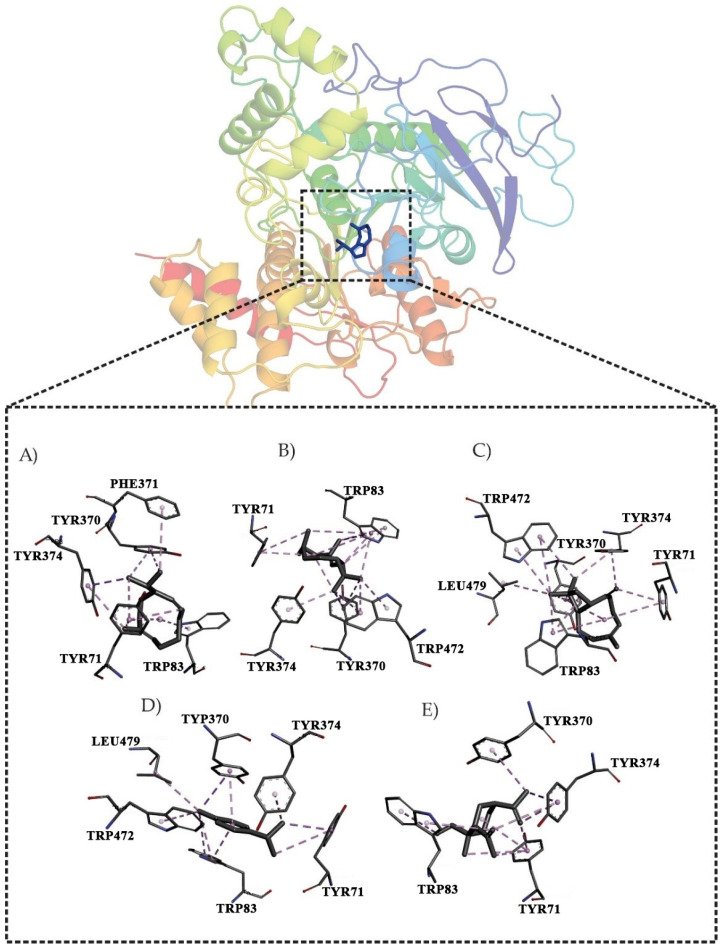
Molecular interactions in the binding pocket of AChE. Germacrene (**A**); bicyclogermacrene (**B**); (*E*)-caryophyllene (**C**); γ-terpinene (**D**); δ-elemene (**E**).

The active site interactions are shown in Figure 1. Germacrene D established π-alkyl interactions with Tyr374, Tyr370, Phe371, Trp83, and Tyr71 (Figure 1A). The ligand bicyclogermacrene formed π-alkyl interactions with Tyr71, Tyr370, Tyr374, Trp472, and Trp83 (Figure 1B). For (*E*)-caryophyllene, the active site formed hydrophobic π-alkyl interactions with Trp472, Leu479, Trp83, Tyr71 Tyr374, and Tyr370 (Figure 1C). γ-Terpinene (8.99%) formed π-alkyl interactions with Tyr71, Tyr374, Trp83, Tyr370, Leu479, and Trp472 (Figure 1D). The binding of δ-elemene to the AChE binding pocket formed π-alkyl hydrophobic interactions with residues Tyr370, Tyr374, Tyr71, and Trp83 (Figure 1D).

## 4. Conclusions

The yield of the *C. campinarensis* EO analyzed in this study was 0.24%. Terpenes characterized the aromatic profile of the EO, with a predominance of sesquiterpene hydrocarbons (87.95%), mainly germacrene D (26.95%), bicyclogermacrene (17.08%), (*E*)-caryophyllene (17.06%), and δ-elemene (7.59%). Regarding the antioxidant capacity of the EO, the evaluation of TEAC by the ABTS method showed that the EO has moderate antioxidant activity. However, the TEAC evaluation showed important inhibition of DPPH^•^ radicals. The preliminary cytotoxicity test against *A. salina* indicated that the EO of *C. campinarensis* can be classified as toxic, with an LC_50_ of 20.84 ± 4.84 µg·mL^−1^. The energy calculations in Table 1 showed that complex formation was favorable. Hydrophobic interactions dominated the interactions between the major compounds of *C. campinarensis* EO and AChE. These results may indicate that the chemical components with higher contents in the sample may be related to the high toxicity demonstrated by the EO against the microcrustacean *A. salina*. This study presents the first report on the chemical composition, antioxidant capacity, and preliminary toxicity of the EO of *C. campinarensis*, contributing to the knowledge of the phytochemistry of species of the genus *Croton* from the Amazon region.

## Figures and Tables

**Table 1:** Main chemical constituents and EO yield of *C. campinarensis*.

Yield (%)			0.24
RI_L_	RI_C_	Chemical Constituents	Area (%)
1014 ^a^	1017	α-terpinene	0.31
1020 ^a^	1024	*p*-cymene	0.49
1054 ^a^	1059	γ-terpinene	8.99
1335 ^a^	1339	δ-elemene	7.59
1374 ^a^	1378	α-copaene	0.34
1387 ^a^	1387	β-bourbonene	0.45
1389 ^a^	1394	β-elemene	3.56
1409 ^a^	1412	α-gurjunene	0.48
1417 ^a^	1423	(*E*)-caryophyllene	17.60
1430 ^a^	1431	β-copaene	1.37
1434 ^a^	1434	γ-elemene	0.43
1432 ^a^	1437	α-*trans*-bergamotene	1.03
1447 ^b^	1446	isogermacrene D	0.60
1452 ^a^	1456	α-humulene	2.49
1458 ^a^	1463	*allo*-aromadrendene	0.38
1465 ^a^	1465	*cis*-muurola-4(14).5-diene	0.21
1478 ^a^	1479	γ-muurolene	0.39
1484 ^a^	1484	germacrene D	26.95
1495 ^a^	1492	γ-amorphene	0.39
1500 ^a^	1500	bicyclogermacrene	17.98
1504 ^a^	1506	cuparene	1.68
1514 ^a^	1513	β-curcumene	0.28
1514 ^a^	1516	(*Z*)-γ-bisabolene	1.01
1522 ^a^	1525	δ-cadinene	1.38
1529 ^a^	1533	(*E*)-γ-bisabolene	1.06
1533 ^a^	1539	*trans*-cadina-1.4-diene	0.09
1537	1544	(*E*)-α-bisabolene	0.21
1577 ^a^	1581	spathulenol	0.96
1582	1587 ^a^	caryophyllene oxide	0.31
1638	1643 ^a^	*epi*-α-cadinol	0.33
1644	1649 ^a^	α-muurolol	0.21
1652	1655 ^a^	α-cadinol	0.31
monoterpene hydrocarbons			9.30
oxygenated monoterpenes			0.00
sesquiterpene hydrocarbons			87.95
oxygenated sesquiterpenes			2.12
other			0.49
TOTAL			99.86

RI_C_, retention index, RI_L_: retention index from the literature: (a) = [52] and (b) = [67]. Relative percentage areas calculated based on the peak areas.

**Table 2:** Trolox equivalent antioxidant capacity of *C. campinarensis* EO for inhibition of ABTS^•+^ and DPPH^•^ radicals and preliminary toxicity against *A. salina*.

TEAC		Preliminary Toxicity	
ABTS (mM·L^−1^)	DPPH (mM·L^−1^)	LC_50_ (µg·mL^−1^)	R^2^
0.55 ± 0.04 ^a^	1.88 ± 0.08 ^b^	20.84 ± 4.84	0.85

Values are expressed as the mean and standard deviation (*n* = 3) of Trolox equivalent antioxidant capacity. Student’s *t*-test was used to compare OE of *C. campinarensis* to the Trolox standard (1 mM·L^−1^). TEAC = Trolox equivalent antioxidant capacity. Different letters indicate that the samples are significantly different.

**Table 3:** Moldock scores obtained from the docking protocol using MVD 5.5.

Molecule	MolDock Score	Rerank Score
Germacrene D	−01.107	−55.75
Bicyclogermacrene	−95.71	−71.41
(*E*)-caryophyllene	−103.70	−80.34
γ-terpinen	−49.42	−43.07
δ-elemene	−89.36	−70.84

## Data Availability

The data are contained within this article.

## References

[1] Rout S., Tambe S., Deshmukh R.K., Mali S., Cruz J., Srivastav P.P., Amin P.D., Gaikwad K.K., de Aguiar Andrade E.H., de Oliveira M.S. (2022). Recent Trends in the Application of Essential Oils: The next Generation of Food Preservation and Food Packaging. Trends Food Sci. Technol..

[2] de Oliveira M.S., de Oliveira M.S. (2022). Essential Oils.

[3] Cascaes M.M., Silva S.G., Cruz J.N., De Oliveira S., Oliveira J., Antonio A., Moraes B.D., Augusto F., Santana K., Diniz L. (2021). First Report on the *Annona exsucca* DC. Essential Oil and *in silico* Identification of Potential Biological Targets of Its Major Compounds. Nat. Prod. Res..

[4] Bezerra F.W.F., de Oliveira M.S., Bezerra P.N., Cunha V.M.B., Silva M.P., da Costa W.A., Pinto R.H.H., Cordeiro R.M., da Cruz J.N., Chaves Neto A.M.J., Inamuddin R.M., Boddula R., Asiri A.M. (2020). Extraction of Bioactive Compounds. Green Sustainable Process for Chemical and Environmental Engineering and Science.

[5] Rodrigues T.L.M., Castro G.L.S., Viana R.G., Gurgel E.S.C., Silva S.G., de Oliveira M.S., de Aguiar Andrade E.H. (2020). Physiological Performance and Chemical Compositions of the *Eryngium foetidum* L. (Apiaceae) Essential Oil Cultivated with Different Fertilizer Sources. Nat. Prod. Res..

[6] Gontijo D.C., do Nascimento M.F.A., Brandão G.C., de Oliveira A.B. (2019). Phytochemistry and Antiplasmodial Activity of Xylopia Sericea Leaves. Nat. Prod. Res..

[7] Cruz J.N., de Oliveira M.S., de Aguiar Andrade E.H., Rodrigues Lima R. (2022). Molecular Modeling Approaches Can Reveal the Molecular Interactions Established between a Biofilm and the Bioactive Compounds of the Essential Oil of *Piper divaricatum*. Molecules.

[8] Maia O.G.S., Andrade L.H.A. (2009). Database of the Amazon Aromatic Plants and Their Essential Oils. Quim. Nova.

[9] Cascaes M.M., Carneiro O.D.S., do Nascimento L.D., de Moraes Â.A.B., de Oliveira M.S., Cruz J.N., Guilhon G.M.S.P., Andrade E.H.D.A. (2021). Essential Oils from Annonaceae Species from Brazil: A Systematic Review of Their Phytochemistry, and Biological Activities. Int. J. Mol. Sci..

[10] Silva S.G., de Oliveira M.S., Cruz J.N., da Costa W.A., da Silva S.H.M., Barreto Maia A.A., de Sousa R.L., Carvalho Junior R.N., de Aguiar Andrade E.H. (2021). Supercritical CO_2_ Extraction to Obtain *Lippia thymoides* Mart. & Schauer (Verbenaceae) Essential Oil Rich in Thymol and Evaluation of Its Antimicrobial Activity. J. Supercrit. Fluids.

[11] Ferreira O.O., da Cruz J.N., Franco C.D.J.P., Silva S.G., da Costa W.A., de Oliveira M.S., Andrade E.H.D.A. (2020). First Report on Yield and Chemical Composition of Essential Oil Extracted from *Myrcia eximia* DC (Myrtaceae) from the Brazilian Amazon. Molecules.

[12] Da Cruz E.D.N.S., Peixoto L.D.S., da Costa J.S., Mourão R.H.V., do Nascimento W.M.O., Maia J.G.S., Setzer W.N., da Silva J.K., Figueiredo P.L.B. (2022). Seasonal Variability of a Caryophyllane Chemotype Essential Oil of *Eugenia patrisii* Vahl Occurring in the Brazilian Amazon. Molecules.

[13] de Lima E.J.S.P., Alves R.G., D’elia G.M.A., da Anunciação T.A., Silva V.R., Santos L.D.S., Soares M.B.P., Cardozo N.M.D., Costa E.V., da Silva F.M.A. (2018). Antitumor Effect of the Essential Oil from the Leaves of *Croton matourensis* Aubl. (Euphorbiaceae). Molecules.

[14] Cucho-Medrano J.L.L., Mendoza-Beingolea S.W., Fuertes-Ruitón C.M., Salazar-Salvatierra M.E., Herrera-Calderon O. (2021). Chemical Profile of the Volatile Constituents and Antimicrobial Activity of the Essential Oils from *Croton adipatus*, *Croton thurifer*, and *Croton collinus*. Antibiotics.

[15] Caruzo M.B.R., Secco R.S., Medeiros D., Riina R., Torres D.S.C., Santos R.F.D., Pereira A.P.N., Rossine Y., Lima L.R., Muniz Filho E. Croton in Flora e Funga Do Brasil. https://floradobrasil.jbrj.gov.br/FB17497.

[16] Guerra Júnior J.I., Ferreira M.R.A., de Oliveira A.M., Soares L.A.L. (2022). Croton Sp.: A Review about Popular Uses, Biological Activities and Chemical Composition. Res. Soc. Dev..

[17] Silva P.M.D.S., Fiaschitello T.R., de Queiroz R.S., Freeman H.S., da Costa S.A., Leo P., Montemor A.F., da Costa S.M. (2020). Natural Dye from *Croton urucurana* Baill. Bark: Extraction, Physicochemical Characterization, Textile Dyeing and Color Fastness Properties. Dye. Pigment..

[18] Ferreira O.O., Cruz J.N., de Moraes Â.A.B., de Jesus Pereira Franco C., Lima R.R., dos Anjos T.O., Siqueira G.M., do Nascimento L.D., Cascaes M.M., de Oliveira M.S. (2022). Essential Oil of the Plants Growing in the Brazilian Amazon: Chemical Composition, Antioxidants, and Biological Applications. Molecules.

[19] Sá Firmino N.C., Alexandre F.S.O., de Vasconcelos M.A., Pinheiro A.A., Arruda F.V.S., Guedes M.L.S., Silveira E.R., Teixeira E.H. (2019). Diterpenes Isolated from *Croton blanchetianus* Baill: Potential Compounds in Prevention and Control of the Oral Streptococci Biofilms. Ind. Crops Prod..

[20] Da Silva Brito S.S., Silva F., Malheiro R., Baptista P., Pereira J.A. (2018). *Croton argyrophyllus* Kunth and *Croton heliotropiifolius* Kunth: Phytochemical Characterization and Bioactive Properties. Ind. Crops Prod..

[21] de Alencar Filho J.M.T., Araújo L.D.C., Oliveira A.P., Guimarães A.L., Pacheco A.G.M., Silva F.S., Cavalcanti L.S., Lucchese A.M., Almeida J.R.G.D.S., Araújo E.C.D.C. (2017). Chemical Composition and Antibacterial Activity of Essential Oil from Leaves of *Croton heliotropiifolius* in Different Seasons of the Year. Rev. Bras. Farmacogn..

[22] Sousa A., Oliveira G., Fonseca L., Rocha M., Rai M., Santos F., de Lima S. (2022). Antioxidant Properties of *Croton zehntneri* Pax et Hoffm. Essential Oil and Its Inclusion Complex with β-Cyclodextrin Prepared by Spray Drying. J. Braz. Chem. Soc..

[23] Coelho-de-Souza A.N., Rocha M.V.A.P., Oliveira K.A., Vasconcelos Y.A.G., Santos E.C., Silva-Alves K.S., Diniz L.R.L., Ferreira-da-Silva F.W., Oliveira A.C., Ponte E.L. (2019). Volatile Oil of *Croton zehntneri* per Oral Sub-Acute Treatment Offers Small Toxicity: Perspective of Therapeutic Use. Rev. Bras. Farmacogn..

[24] de Souza G.S., Bonilla O.H., de Lucena E.M.P., Barbosa Y.P. (2017). Rendimento e Composição Química Do Óleo Essencial de Três Espécies de Croton. Cienc. Rural.

[25] Lima C.C., de Holanda-Angelin-Alves C.M., Pereira-Gonçalves Á., Kennedy-Feitosa E., Evangelista-Costa E., Bezerra M.A.C., Coelho-de-Souza A.N., Leal-Cardoso J.H. (2020). Antispasmodic Effects of the Essential Oil of *Croton zehnteneri*, Anethole, and Estragole, on Tracheal Smooth Muscle. Heliyon.

[26] Rocha R.R., Matos M.N.C., Guerrero J.A.P., Cavalcante R.M.B., Melo R.S., Azevedo Á.M.A., Pereira A.M.G., Lopes P.H.R., Rodrigues T.H.S., Bandeira P.N. (2021). Comparative Study of the Chemical Composition, Antibacterial Activity and Synergic Effects of the Essential Oils of *Croton tetradenius* Baill. And *C. pulegiodorus* Baill. Against *Staphylococcus aureus* Isolates. Microb. Pathog..

[27] Almeida J., Souza A.V., Oliveira A.P., Santos U., Souza M., Bispo L., Turatti Z.C., Lopes N. (2014). Chemical Composition of Essential Oils from *Croton conduplicatus* (Euphorbiaceae) in Two Different Seasons. J. Essent. Oil Bear. Plants.

[28] Azevedo M.M.B., Chaves F.C.M., Almeida C.A., Bizzo H.R., Duarte R.S., Campos-Takaki G.M., Alviano C.S., Alviano D.S. (2013). Antioxidant and Antimicrobial Activities of 7-Hydroxycalamenene-Rich Essential Oils from *Croton cajucara* Benth. Molecules.

[29] Donati M., Mondin A., Chen Z., Miranda F.M., Do Nascimento B.B., Schirato G., Pastore P., Froldi G. (2015). Radical Scavenging and Antimicrobial Activities of *Croton zehntneri*, *Pterodon emarginatus* and *Schinopsis brasiliensis* Essential Oils and Their Major Constituents: Estragole, Trans -Anethole, β-Caryophyllene and Myrcene. Nat. Prod. Res..

[30] Souto E.B., Severino P., Marques C., Andrade L.N., Durazzo A., Lucarini M., Atanasov A.G., El Maimouni S., Novellino E., Santini A. (2020). *Croton argyrophyllus* Kunth Essential Oil-Loaded Solid Lipid Nanoparticles: Evaluation of Release Profile, Antioxidant Activity and Cytotoxicity in a Neuroblastoma Cell Line. Sustainability.

[31] Simionatto E., Bonani V.F.L., Morel A.F., Poppi N.R., Raposo Júnior J.L., Stuker C.Z., Peruzzo G.M., Peres M.T.L.P., Hess S.C. (2007). Chemical Composition and Evaluation of Antibacterial and Antioxidant Activities of the Essential Oil of *Croton urucurana* Baillon (Euphorbiaceae) Stem Bark. J. Braz. Chem. Soc..

[32] Ramos da Silva L.R., Ferreira O.O., Cruz J.N., de Jesus Pereira Franco C., dos Anjos T.O., Cascaes M.M., Almeida da Costa W., Helena de Aguiar Andrade E., Santana de Oliveira M. (2021). Lamiaceae Essential Oils, Phytochemical Profile, Antioxidant, and Biological Activities. Evid. Based Complement. Altern. Med..

[33] Franco C.D.J.P., Ferreira O.O., de Moraes Â.A.B., Varela E.L.P., do Nascimento L.D., Percário S., de Oliveira M.S., Andrade E.H.D.A. (2021). Chemical Composition and Antioxidant Activity of Essential Oils from *Eugenia patrisii* Vahl, *E. punicifolia* (Kunth) DC., and *Myrcia tomentosa* (Aubl.) DC., Leaf of Family Myrtaceae. Molecules.

[34] Diniz Do Nascimento L., De Moraes A.A.B., Da Costa K.S., Marcos J., Galúcio P., Taube P.S., Leal Costa M., Neves Cruz J., De Aguiar Andrade E.H., De Faria L.J.G. (2020). Bioactive Natural Compounds and Antioxidant Activity of Essential Oils from Spice Plants: New Findings and Potential Applications. Biomolecules.

[35] Mutlu-Ingok A., Devecioglu D., Dikmetas D.N., Karbancioglu-Guler F., Capanoglu E. (2020). Antibacterial, Antifungal, Antimycotoxigenic, and Antioxidant Activities of Essential Oils: An Updated Review. Molecules.

[36] do Nascimento L.D., Silva S.G., Cascaes M.M., da Costa K.S., Figueiredo P.L.B., Costa C.M.L., Andrade E.H.D.A., de Faria L.J.G. (2021). Drying Effects on Chemical Composition and Antioxidant Activity of Lippia Thymoides Essential Oil, a Natural Source of Thymol. Molecules.

[37] de Oliveira M.S., Silva S.G., da Cruz J.N., Ortiz E., da Costa W.A., Bezerra F.W.F., Cunha V.M.B., Cordeiro R.M., de Jesus Chaves Neto A.M., de Andrade E.H.A., Inamuddin R.M., Asiri A.M. (2019). Supercritical CO_2_ Application in Essential Oil Extraction. Industrial Applications of Green Solvents—Volume II..

[38] Fuentes C., Fuentes A., Barat J.M., Ruiz M.J. (2021). Relevant Essential Oil Components: A Minireview on Increasing Applications and Potential Toxicity. Toxicol. Mech. Methods.

[39] da Silva Júnior O.S., de Jesus Pereira Franco C., de Moraes A.A.B., Cruz J.N., da Costa K.S., Diniz do Nascimento L., de Aguiar Andrade E.H. (2021). In Silico Analyses of Toxicity of the Major Constituents of Essential Oils from Two *Ipomoea* L. Species. Toxicon.

[40] Oliva M.D.L.M., Gallucci N., Zygadlo J.A., Demo M.S. (2007). Cytotoxic Activity of Argentinean Essential Oils on *Artemia salina*. Pharm. Biol..

[41] Brasil D.D.S.B., Muller A.H., Guilhon G.M.S.P., Alves C.N., Andrade E.H.A., da Silva J.K.R., Maia J.G.S. (2009). Essential Oil Composition of *Croton palanostigma* Klotzsch from North Brazil. J. Braz. Chem. Soc..

[42] Da Costa J.G.M., Rodrigues F.F.G., Angélico E.C., Pereira C.K.B., De Souza E.O., Caldas G.F.R., Silva M.R., Santos N.K.A., Mota M.L., Dos Santos P.F. (2008). Chemical Composition and Evaluation of the Antibacterial Activity and Toxicity of the Essential Oil of *Croton zehntneri* (Variety Estragol). Rev. Bras. Farmacogn..

[43] De Lima S.G., Medeiros L.B.P., Cunha C.N.L.C., Da Silva D., De Andrade N.C., Moita Neto J.M., Lopes J.A.D., Steffen R.A., Araújo B.Q., De Reis F.A.M. (2012). Chemical Composition of Essential Oils of *Croton hirtus* L’Her from Piauí (Brazil). J. Essent. Oil Res..

[44] Werka J.S., Boehme A.K., Setzer W.N. (2007). Biological Activities of Essential Oils from Monteverde, Costa Rica. Nat. Prod. Commun..

[45] Mesquita K.D.S.M., Feitosa B.D.S., Cruz J.N., Ferreira O.O., Franco C.D.J.P., Cascaes M.M., de Oliveira M.S., Andrade E.H.D.A. (2021). Chemical Composition and Preliminary Toxicity Evaluation of the Essential Oil from *Peperomia circinnata* Link Var. circinnata. (Piperaceae) in Artemia salina Leach. Molecules.

[46] Baek I., Choi H.J., Rhee J.S. (2015). Inhibitory Effects of Biocides on Hatching and Acetylcholinesterase Activity in the Brine Shrimp *Artemia salina*. Toxicol. Environ. Health Sci..

[47] Secco R.D.S., Rosário A.S.D., Berry P.E. (2012). *Croton campinarensis* (Euphorbiaceae), a New Species from Eastern Amazonian Brazil. Phytotaxa.

[48] Ferreira O.O., da Silva S.H.M., de Oliveira M.S., Andrade E.H.D.A. (2021). Chemical Composition and Antifungal Activity of Myrcia Multiflora and Eugenia Florida Essential Oils. Molecules.

[49] de Oliveira M.S., da Cruz J.N., da Costa W.A., Silva S.G., Brito M.D.P., de Menezes S.A.F., de Jesus Chaves Neto A.M., de Aguiar Andrade E.H., de Carvalho Junior R.N. (2020). Chemical Composition, Antimicrobial Properties of Siparuna Guianensis Essential Oil and a Molecular Docking and Dynamics Molecular Study of Its Major Chemical Constituent. Molecules.

[50] Ferreira O.O., Franco C.D.J.P., Varela E.L.P., Silva S.G., Cascaes M.M., Percário S., de Oliveira M.S., Andrade E.H.D.A. (2021). Chemical Composition and Antioxidant Activity of Essential Oils from Leaves of Two Specimens of Eugenia Florida DC. Molecules.

[51] van Den Dool H., Kratz P.D. (1963). A Generalization of the Retention Index System Including Linear Temperature Programmed Gas—Liquid Partition Chromatography. J. Chromatogr. A.

[52] Adams R.P. (2007). Identification of Essential Oil Components by Gas Chromatography/Mass Spectrometry.

[53] Blois M.S. (1958). Antioxidant Determinations by the Use of a Stable Free Radical. Nature.

[54] de Moraes Â.A.B., Ferreira O.O., da Costa L.S., Almeida L.Q., Varela E.L.P., Cascaes M.M., de Jesus Pereira Franco C., Percário S., do Nascimento L.D., de Oliveira M.S. (2022). Phytochemical Profile, Preliminary Toxicity, and Antioxidant Capacity of the Essential Oils of *Myrciaria floribunda* (H. West Ex Willd.) O. Berg. and *Myrcia sylvatica* (G. Mey) DC. (Myrtaceae). Antioxidants.

[55] Re R., Pellegrini N., Proteggente A., Pannala A., Yang M., Rice-Evans C. (1999). Antioxidant Activity Applying an Improved ABTS Radical Cation Decolorization Assay. Free Radic. Biol. Med..

[56] Miller N.J., Rice-Evans C., Davies M.J., Gopinathan V., Milner A. (1993). A Novel Method for Measuring Antioxidant Capacity and Its Application to Monitoring the Antioxidant Status in Premature Neonates. Clin. Sci..

[57] Neto R.D.A.M., Santos C.B.R., Henriques S.V.C., Machado L.D.O., Cruz J.N., da Silva C.H.T.D.P., Federico L.B., de Oliveira E.H.C., de Souza M.P.C., da Silva P.N.B. (2022). Novel Chalcones Derivatives with Potential Antineoplastic Activity Investigated by Docking and Molecular Dynamics Simulations. J. Biomol. Struct. Dyn..

[58] Rego C.M.A., Francisco A.F., Boeno C.N., Paloschi M.V., Lopes J.A., Silva M.D.S., Santana H.M., Serrath S.N., Rodrigues J.E., Lemos C.T.L. (2022). Inflammasome NLRP3 Activation Induced by Convulxin, a C-Type Lectin-like Isolated from *Crotalus durissus terrificus* Snake Venom. Sci. Rep..

[59] Almeida V.M., Dias Ê.R., Souza B.C., Cruz J.N., Santos C.B.R., Leite F.H.A., Queiroz R.F., Branco A. (2021). Methoxylated Flavonols from Vellozia Dasypus Seub Ethyl Acetate Active Myeloperoxidase Extract: In Vitro and in Silico Assays. J. Biomol. Struct. Dyn..

[60] Lima A.D.M., Siqueira A.S., Möller M.L.S., Souza R.C.D., Cruz J.N., Lima A.R.J., da Silva R.C., Aguiar D.C.F., Junior J.L., Gonçalves E.C. (2020). In Silico Improvement of the Cyanobacterial Lectin Microvirin and Mannose Interaction. J. Biomol. Struct. Dyn..

[61] Thomsen R., Christensen M.H. (2006). MolDock: A New Technique for High-Accuracy Molecular Docking. J. Med. Chem..

[62] Leão R.P., Cruz J.V., da Costa G.V., Cruz J.N., Ferreira E.F.B., Silva R.C., de Lima L.R., Borges R.S., dos Santos G.B., Santos C.B.R. (2020). Identification of New Rofecoxib-Based Cyclooxygenase-2 Inhibitors: A Bioinformatics Approach. Pharmaceuticals.

[63] Mascarenhas A.M.S., de Almeida R.B.M., de Araujo Neto M.F., Mendes G.O., da Cruz J.N., dos Santos C.B.R., Botura M.B., Leite F.H.A. (2020). Pharmacophore-Based Virtual Screening and Molecular Docking to Identify Promising Dual Inhibitors of Human Acetylcholinesterase and Butyrylcholinesterase. J. Biomol. Struct. Dyn..

[64] Santos C.B.R., Santos K.L.B., Cruz J.N., Leite F.H.A., Borges R.S., Taft C.A., Campos J.M., Silva C.H.T.P. (2021). Molecular Modeling Approaches of Selective Adenosine Receptor Type 2A Agonists as Potential Anti-Inflammatory Drugs. J. Biomol. Struct. Dyn..

[65] Cheung J., Gary E.N., Shiomi K., Rosenberry T.L. (2013). Structures of Human Acetylcholinesterase Bound to Dihydrotanshinone i and Territrem B Show Peripheral Site Flexibility. ACS Med. Chem. Lett..

[66] Turiel N.A., Ribeiro A.F., Carvalho N.C.C., Monteiro O.S., Lucas F.C.A., Carreira L.M.M., Andrade E.H.A., Maia J.G.S. (2016). Variability in Essential Oil Composition of Croton Species with Occurrence in the Eastern Brazilian Amazon. Rec. Nat. Prod..

[67] Stein S., Mirokhin D., Tchekhovskoi D., Mallard G., Mikaia A., Zaikin V., Sparkmanm D. (2011). Standard Reference Data Program of the National Institute of Standards and Technology.

[68] Albuquerque B.N.D.L., Da Silva M.F.R., Da Silva P.C.B., De Lira Pimentel C.S., Lino Da Rocha S.K., De Aguiar J.C.R.O.F., Neto A.C.A., Paiva P.M.G., Gomes M.G.M., Da Silva-Júnior E.F. (2022). Oviposition Deterrence, Larvicidal Activity and Docking of β-Germacrene-D-4-Ol Obtained from Leaves of *Piper corcovadensis* (Piperaceae) against *Aedes aegypti*. Ind. Crops Prod..

[69] Formagio A.S.N., Vilegas W., Volobuff C.R.F., Kassuya C.A.L., Cardoso C.A.L., Pereira Z.V., Silva R.M.M.F., dos Santos Yamazaki D.A., de Freitas Gauze G., Manfron J. (2022). Exploration of Essential Oil from Psychotria Poeppigiana as an Anti-Hyperalgesic and Anti-Acetylcholinesterase Agent: Chemical Composition, Biological Activity and Molecular Docking. J. Ethnopharmacol..

[70] Francomano F., Caruso A., Barbarossa A., Fazio A., La Torre C., Ceramella J., Mallamaci R., Saturnino C., Iacopetta D., Sinicropi M.S. (2019). β-Caryophyllene a Sesquiterpene with Countless. Appl. Sci..

[71] Reis J.B., Figueiredo L.A., Castorani G.M., Veiga S.M.O.M. (2020). Avaliação Da Atividade Antimicrobiana Dos Óleos Essenciais Contra Patógenos Alimentares. Braz. J. Health Rev..

[72] Lu J.J., Dang Y.Y., Huang M., Xu W.S., Chen X.P., Wang Y.T. (2012). Anti-Cancer Properties of Terpenoids Isolated from Rhizoma Curcumae—A Review. J. Ethnopharmacol..

[73] Da Silva Júnior O.S., Franco C.D.J.P., de Moraes Â.A.B., Pastore M., Cascaes M.M., do Nascimento L.D., de Oliveira M.S., Andrade E.H.D.A. (2022). Chemical Variability of Volatile Concentrate from Two *Ipomoea* L. Species within a Seasonal Gradient. Nat. Prod. Res..

[74] Ramos S.C.S., De Oliveira J.C.S., Da Câmara C.A.G., Castelar I., Carvalho A.F.F.U., Lima-Filho J.V. (2009). Antibacterial and Cytotoxic Properties of Some Plant Crude Extracts Used in Northeastern Folk Medicine. Rev. Bras. Farmacogn..

[75] Morais S.M., Cossolosso D.S., Silva A.A.S., de Moraes Filho M.O., Teixeira M.J., Campello C.C., Bonilla O.H., de Paula V.F., Vila-Nova N.S. (2019). Essential Oils from Croton Species: Chemical Composition, in Vitro and in Silico Antileishmanial Evaluation, Antioxidant and Cytotoxicity Activities. J. Braz. Chem. Soc..

[76] Pino J.A., Terán-Portelles E.C., Hernández I., Rodeiro I., Fernández M.D. (2018). Chemical Composition of the Essential Oil from *Croton wagneri* Müll. Arg. (Euphorbiaceae) Grown in Ecuador. J. Essent. Oil Res..

[77] Do Vale J.P.C., Vasconcelos M.A., Arruda F.V.S., Firmino N.C.S., Pereira A.L., Andrade A.L., Saker-Sampaio S., Sampaio A.H., Marinho E.S., Teixeira A.M.R. (2021). Evaluation of Antimicrobial and Antioxidant Potential of Essential Oil from *Croton piauhiensis* Müll. Arg. Curr. Microbiol..

[78] Ribeiro L.P., Domingues V.C., Gonçalves G.L.P., Fernandes J.B., Glória E.M., Vendramim J.D. (2020). Essential Oil from *Duguetia lanceolata* St.-Hil. (Annonaceae): Suppression of Spoilers of Stored-Grain. Food Biosci..

[79] Andrade T.C.B., Lima S.G.D., Freitas R.M., Rocha M.S., Islam T., Silva T.G.D., Militão G.C.G. (2015). Isolation, Characterization and Evaluation of Antimicrobial and Cytotoxic Activity of Estragole, Obtained from the Essential Oil of *Croton zehntneri* (Euphorbiaceae). An. Da Acad. Bras. De Ciências.

[80] Lawal O.A., Ogunwande I.A., Osunsanmi F.O., Opoku A.R., Oyedeji A.O. (2017). *Croton gratissimus* Leaf Essential Oil Composition, Antibacterial, Antiplatelet Aggregation, and Cytotoxic Activities. J. Herbs Spices Med. Plants.

[81] Ascari J., de Oliveira M.S., Nunes D.S., Granato D., Scharf D.R., Simionatto E., Otuki M., Soley B., Heiden G. (2019). Chemical Composition, Antioxidant and Anti-Inflammatory Activities of the Essential Oils from Male and Female Specimens of *Baccharis punctulata* (Asteraceae). J. Ethnopharmacol..

[82] Casiglia S., Bruno M., Bramucci M., Quassinti L., Lupidi G., Fiorini D., Maggi F. (2017). *Kundmannia sicula* (L.) DC: A Rich Source of Germacrene D. J. Essent. Oil Res..

[83] Dahham S.S., Tabana Y.M., Iqbal M.A., Ahamed M.B.K., Ezzat M.O., Majid A.S.A., Majid A.M.S.A. (2015). The Anticancer, Antioxidant and Antimicrobial Properties of the Sesquiterpene β-Caryophyllene from the Essential Oil of Aquilaria Crassna. Molecules.

[84] de Castro J.A.M., Monteiro O.S., Coutinho D.F., Rodrigues A.A.C., da Silva J.K.R., Maia J.G.S. (2019). Seasonal and Circadian Study of a Thymol/γ-Terpinene/p-Cymene Type Oil of *Ocimum gratissimum* L. And Its Antioxidant and Antifungal Effects. J. Braz. Chem. Soc..

[85] Zuccolotto T., Bressan J., Lourenço A.V.F., Bruginski E., Veiga A., Marinho J.V.N., Raeski P.A., Heiden G., Salvador M.J., Murakami F.S. (2019). Chemical, Antioxidant, and Antimicrobial Evaluation of Essential Oils and an Anatomical Study of the Aerial Parts from *Baccharis* Species (Asteraceae). Chem. Biodivers..

[86] Guo Y., Liu Z., Hou E., Ma N., Fan J., Jin C.Y., Yang R. (2020). Non-Food Bioactive Natural Forest Products as Insecticide Candidates: Preparation, Biological Evaluation and Molecular Docking Studies of Novel N-(1,3-Thiazol-2- Yl)Carboxamides Fused (+)-Nootkatone from Chamaecyparis Nootkatensis [D. Don] Spach. Ind. Crops Prod..

[87] Mollica F., Gelabert I., Amorati R. (2022). Synergic Antioxidant Effects of the Essential Oil Component γ-Terpinene on High-Temperature Oil Oxidation. ACS Food Sci. Technol..

[88] Judžentienė A., Būdienė J. (2021). Mugwort (*Artemisia vulgaris* L.) Essential Oils Rich in Germacrene D, and Their Toxic Activity. J. Essent. Oil Res..

[89] Machado K.D.C., Paz M.F.C.J., de Oliveira Santos J.V., da Silva F.C.C., Tchekalarova J.D., Salehi B., Islam M.T., Setzer W.N., Sharifi-Rad J., de Castro e Sousa J.M. (2020). Anxiety Therapeutic Interventions of β-Caryophyllene: A Laboratory-Based Study. Nat. Prod. Commun..

[90] Schmitt D., Levy R., Carroll B. (2016). Toxicological Evaluation of β-Caryophyllene Oil: Subchronic Toxicity in Rats. Int. J. Toxicol..

[91] Govindarajan M., Rajeswary M., Hoti S.L., Bhattacharyya A., Benelli G. (2016). Eugenol, α-Pinene and β-Caryophyllene from *Plectranthus barbatus* Essential Oil as Eco-Friendly Larvicides against Malaria, Dengue and Japanese Encephalitis Mosquito Vectors. Parasitol. Res..

[92] De Oliveira M.R.C., Barros L.M., Duarte A.E., De Lima Silva M.G., Da Silva B.A.F., Pereira Bezerra A.O.B., Oliveira Tintino C.D.M., De Oliveira V.A.P., Boligon A.A., Kamdem J.P. (2019). Gc-Ms Chemical Characterization and in Vitro Evaluation of Antioxidant and Toxic Effects Using Drosophila Melanogaster Model of the Essential Oil of *Lantana montevidensis* (Spreng) Briq. Medicina.

[93] Ojah E.O., Moronkola D.O., Petrelli R., Nzekoue F.K. (2019). Chemical Composition of Ten Essential Oils from Calophyllum Inophyllum Linn and Their Toxicity Against *Artemia salina*. Eur. J. Pharm. Med. Res..

[94] Fabri N.T., Gatto L.J., Furusho A.S., Garcia M.J.B., Marques F.D.A., Miguel M.D., Montrucchio D.P., Zanin S.M.W., Miguel O.G., Gaspari Dias J.D.F. (2019). Composition, Antioxidant Properties, and Biological Activities of the Essential Oil Extracted from *Ocotea diospyrifolia* (Meisn.) Mez. Braz. J. Pharm. Sci..

[95] Cho T.J., Park S.M., Yu H., Seo G.H., Kim H.W., Kim S.A., Rhee M.S. (2020). Recent Advances in the Application of Antibacterial Complexes Using Essential Oils. Molecules.

[96] Wojtunik-Kulesza K.A. (2022). Toxicity of Selected Monoterpenes and Essential Oils Rich in These Compounds. Molecules.

[97] Iannone M., Ovidi E., Vitalini S., Laghezza Masci V., Iriti M., Tiezzi A., Garzoli S., Marianelli A. (2022). From Hops to Craft Beers: Production Process, VOCs Profile Characterization, Total Polyphenol and Flavonoid Content Determination and Antioxidant Activity Evaluation. Processes.

[98] dos Santos K.L.B., Cruz J.N., Silva L.B., Ramos R.S., Neto M.F.A., Lobato C.C., Ota S.S.B., Leite F.H.A., Borges R.S., da Silva C.H.T.P. (2020). Identification of Novel Chemical Entities for Adenosine Receptor Type 2a Using Molecular Modeling Approaches. Molecules.

[99] Pinto V.D.S., Araújo J.S.C., Silva R.C., da Costa G.V., Cruz J.N., Neto M.F.D.A., Campos J.M., Santos C.B.R., Leite F.H.A., Junior M.C.S. (2019). In Silico Study to Identify New Antituberculosis Molecules from Natural Sources by Hierarchical Virtual Screening and Molecular Dynamics Simulations. Pharmaceuticals.

[100] Costa E.B., Silva R.C., Espejo-Román J.M., Neto M.F.D.A., Cruz J.N., Leite F.H.A., Silva C.H.T.P., Pinheiro J.C., Macêdo W.J.C., Santos C.B.R. (2020). Chemometric Methods in Antimalarial Drug Design from 1,2,4,5-Tetraoxanes Analogues. SAR QSAR Environ. Res..

[101] Galucio N.C.D.R., Moysés D.D.A., Pina J.R.S., Marinho P.S.B., Gomes Júnior P.C., Cruz J.N., Vale V.V., Khayat A.S., Marinho A.M.D.R. (2022). Antiproliferative, Genotoxic Activities and Quantification of Extracts and Cucurbitacin B Obtained from *Luffa operculata* (L.) Cogn. Arab. J. Chem..

